# The “most beautiful place” where “it’s not possible to live”: A qualitative study of relational well-being in an area of climate vulnerability, Bangladesh

**DOI:** 10.1371/journal.pone.0325972

**Published:** 2025-09-04

**Authors:** Kyra Lilier, Sarah L. Dalglish, Mark Donald C. Reñosa, Samiya A. Selim, Syed Tauheed Raihan, Rafia Islam, Jennifer Das, Ina Danquah, Rainer Sauerborn, Kate Bärnighausen

**Affiliations:** 1 Faculty of Medicine and University Hospital, Heidelberg Institute of Global Health (HIGH), Heidelberg University, Heidelberg, Germany; 2 Dept of International Health, Johns Hopkins School of Public Health, Baltimore, Maryland, United States of America; 3 Institute for Global Health, University College London, London, England; 4 Centre for Sustainable Development, University of Liberal Arts (ULAB), Dhaka, Bangladesh; Indian Research Academy, INDIA

## Abstract

**Purpose:**

Climate change is the greatest global health threat of the 21^st^ century, but little is known about well-being in climate vulnerable populations. We investigate how well-being is shaped by common and unique stressors in an area of climate vulnerability in Bangladesh.

**Methods:**

We present findings from 60 semi-structured in-depth interviews. We inductively analyzed our data following a Reflexive Thematic Analysis approach and then applied a Relational Well-being (RWB) framework.

**Results:**

We found that well-being was influenced negatively by factors such as financial worries, forced migration, social pressure, and natural disasters. Well-being was influenced positively by factors such as financial satisfaction, voluntary migration, social support, and place attachment.

**Conclusions:**

Using relational well-being as a conceptual lens allowed us to explore the dynamism and complexity of factors shaping well-being that were partly specific to the local context and partly rooted in wider societal and global structures. Policies which aim to improve the well-being of climate vulnerable populations should consider relational well-being as a conceptual tool to leverage locally available informal resources, such as suppotive reciprocal relationships.

## Introduction

Climate change stands as the greatest global health threat of the 21^st^ century [1]. While its adverse physical health consequences have received increased attention, the profound toll on mental well-being – especially among populations enduring the harshest climate disruptions – remains critically overlooked [2–4]. As large population groups are predicted to face the adverse effects of a changing climate, understanding the implications and consequences on mental health and human well-being is essential.

The mental health effects of climate change are broadly categorized into direct and indirect outcomes [5,6]. Direct outcomes stem from immediate exposure to extreme weather events – such as floods, storms, heatwaves and wildfires – and are linked to post-traumatic stress disorders, depression, and anxiety [5]. Heat waves and flooding have also been associated with aggressive behavior [5] and increase suicide risk [5,7]. Indirect outcomes, resulting from the gradual disruption of natural and social environments, loss of homes and livelihoods, or climate-related physical illnesses, are more complex and less understood [6]. These include emerging mental conditions like solastalgia – the grief caused by environmental degradation – and eco-anxiety, the chronic fear of environmental uncertainties [6]. Additionally, economic and non-economic losses, limited access to mental health care and disruptions in protective social relationships and community cohesion contribute to rising rates of depression, suicide, and overall decreased emotional well-being [5,6].

Globally, Bangladesh stands at the forefront of this crisis, with one of the most at-risk populations suffering from the consequences of climate change [8]. Rising sea levels, land erosion, intensified storms and extreme heat not only threaten physical health [8–10], but also erode mental well-being. Multiple, interrelated factors undermine health and resilience in Bangladesh – some of which are uncommon in regions less affected by climate change – such as environmental and economic shocks [11], loss of land [12], the breakdown of social relationships and identity [13], constant migration flows [14,15], and limited access to (mental) health and education services [16,17]. Although Bangladesh’s Health-National Adaptation Plan (HNAP) acknowledges mental health and ‘psycho-social well-being’ [18] as critical concerns, it remains a low priority in national health strategies [18]. The scarcity of mental health data [19], coupled with a lack of culturally-sensitive approaches [17], low mental health literacy, and pervasive stigma further hampers research and support efforts [19]. However, evidence suggests that mental well-being in Bangladesh – and across Asia – is closely rooted in strong social networks [16]. This insight aligns with global calls for more collective, community-centered approaches to mental health, rather than solely individual-focused interventions [20,21]. The HNAP’s inclusion of ‘psycho-social well-being’ reflects this shift, and scholars argue that adopting a broader well-being framework may be “more effective and appropriate than a ‘western’ framing of mental illness” [3]. Researchers have started to explore relational well-being in Bangladesh [22], including perspectives of non-economic losses [23].

Well-being offers a powerful framework for understanding and improving mental health in vulnerable communities [14,22,24]. Well-being is defined as including “the presence of positive emotions and moods […], the absence of negative emotions […], satisfaction with life, fulfilment and positive functioning” [25]. Well-being captures “how people *feel* and how they *function* [sense of competence, sense of being connected], both on a personal and a social level, and how people *evaluate* their lives as a whole” [26]. This holistic, positive and person-centered concept emphasizes what matters to people, focusing on opportunities rather than deficiencies and valuing individual perspectives [27]. Recognizing its potential, many global policies have begun to prioritize well-being as a key societal goal [28–32]. The UN Sustainable Development Goals [28], for example, highlight well-being as essential for fostering societal progress beyond economic growth, particularly in countries where basic material needs are met [30,32–34]. The concept of well-being is also promoted as an important approach in contemporary (mental) health research [3,10,35–38] and has been acknowledged as a key concept to engage and support communities and individuals in leading healthy lives [10,39].

While the concept of well-being has gained significant traction in shaping policies and research in high income countries [34], it remains underutilized in LMICs – particularly those on the frontlines of climate change like Bangladesh [27]. Despite its promise as a holistic and culturally sensitive framework, little is known about how well-being is influenced and shaped within these vulnerable contexts. The potential of well-being as a public mental health approach to support communities most affected by climate change remains to be understood. We aim to qualitatively explore the well-being of populations living in one of the most climate vulnerable areas, Bhola Island in Bangladesh, to understand how human well-being is shaped by a changing climate and to identify ways to inform context-specific strategies to support well-being and adaptation.

## Methods

### Study design and setting

We employed a qualitative case study design [40] using semi-structured in-depth interviews to explore well-being in Bhola, a semi-rural island in the Bay of Bengal with approximately 1.7 million inhabitants. Bhola lays within the delta of two large rivers, the Meghna and Tetulia River [41]. The second largest city is Char Fasson (42,000 inhabitants, 2011) [42]. Agriculture and fishery are the main sources of income, while other sectors like public services are developing [41]. Over 96% of the population are Muslim; education levels are low with a literacy rate of 43% and poverty levels and population density are high [41]. Bhola is extremely vulnerable to hydro-meteorological disasters such as cyclones, river erosion, floods and heavy rainfall [43,44]. It is amongst the four most at risk areas in Bangladesh for flooding, cyclones and tidal saline intrusion with great numbers of household experiencing hazard-related illnesses [45]. Despite some recent improvements, disaster preparedness, early warning systems and disaster support are still insufficient [46]. Different forms of migration are common in Bhola, including seasonal, permanent, circular and international migration [47]. Many people have moved out of Bhola, mostly to large cities like Dhaka, where the so-called Bhola slum was built [48]. In the Covid-19 pandemic many migrants returned to their home villages because work opportunities shrank due to lockdowns [49]. Access to health services in Bhola is limited due to underdeveloped facilities and challenging transportation [50]. While evidence relating to the health impacts of climate change in Bhola island is scarce, research from other coastal regions in Bangladesh emphasizes health effects related to water. Health hazards following cyclones include mental trauma, injury, death and increased incidences of infectious diseases, especially water-borne diseases like diarrhea, skin diseases or typhoid fever [51]. Salinization of drinking water due to sea level rise, storms and floods threaten safe water sources and lead to infectious diseases. Additionally, higher salt intake can aggravate hypertension and stroke risk and negatively affect food security [52,53]. Studies that do not specifically focus on coastal areas describe health outcomes that are likely to also affect Bhola residents. Direct health impacts following extreme weather events such as storms or heat waves include psycho-social effects, damage to healthcare infrastructure and increased heat-related stress, morbidity and mortality [10]. Indirect effects comprise water-related vector-borne diseases (e.g., Dengue) [54] or zoonotic diseases [10]. Psycho-social effects encompass depression, anxiety, post-traumatic stress, suicidal ideation and long-term disruption of well-being [3].

More holistic perspectives on health and well-being outcomes are less understood in coastal populations of Bangladesh with studies on well-being mostly focusing on material aspects [55,56]. However, some work exists that builds on more holistic approaches to well-being, namely on the framework of material, relational and subjective well-being by the working group ‘Wellbeing in developing countries’ [27] developed further by White to the Relational Well-being Framework (RWB) [34]. For example, a study on nomadic fisheries finds that financial security, identity and identification with traditions, and strong bonds within the community contribute to well-being, and highlights how the well-being of the nomads is entangled with the well-being of the wider society [57]. Another study on farmers in coastal Bangladesh discovers agency and autonomy, social pressure, fears for future, trust in institutions and the community as factors contributing to well-being [22]. Hoque et al. emphasize the importance of collective identity as farmers and relational understandings of happiness both with regard to the environment and the community for coastal populations in Bangladesh [22].

### Study population and data collection

We purposefully selected men and women aged 18–85 from different community points in Bhola, such as markets or village centeres, who could share various experiences of their life in Bhola. We aimed to create a heterogenous sample with broad socio-demographic characteristics (Table 1). We collected n = 60 interviews to ensure data saturation [58].

**Table 1:** Socio-demographic characteristics of the study population.

Characteristic	Number (%)
Female	20 (33)
Male	40 (67)
Age group	
18-29 Years	22 (37)
30-49 Years	19 (32)
50-69 Years	15 (25)
70 + Years	4 (7)
Occupation (more than 1 possible)	
Housework	18 (30)
Fishing	16 (27)
Farming	9 (15)
Vending/ Commerce	8 (13)
Hotel/ Restaurant	5 (8)
Day Labor	3 (5)
Rickshaw Driving	2 (3)
Other	6 (10)
Religion	
Muslim	58 (97)
Not disclosed	2 (3)
Relationship status	
Married	50 (83)
Single	7 (12)
Partner, not living together	1 (2)
Not disclosed	2 (3)
Number of children	
0–2	27 (45)
3–5	21 (35)
more than 5	12 (20)
Highest education level	
None	25 (42)
Primary Education	21 (35)
High School Education	7 (12)
Tertiary Education	5 (8)
Not disclosed	2 (3)
Total	60 (100)

We trained five Bangladeshi research assistants (RAs), two women and three men, in qualitative data collection via an online platform. RAs were fluent in English and Bangla, were educated to a post-graduate level and were working as RAs at the University in Dhaka. From the 12^th^ to the 17^th^ of October 2020, each RA conducted and audio-recorded two semi-structured in-depth in-person interviews per day using our data collection instruments. These instruments included a participant information sheet with Covid-19 safety information, an informed consent form, and an interview guide available in both Bangla and English (see Text S1 in S1 File). Cover sheets were used to capture socio-demographic data (including sex, age, employment status, children, relationship status and religion) and reflexive and observational notes made by the RAs. Interviews lasted approximately 30–45 minutes. RAs were tested for Covid-19 before leaving for and returning from Bhola, and interviews were conducted outside, socially distanced, with face masks. Prior to the interviews, RAs explained the purpose of the study, obtained written informed consent, and built rapport via informal discussions with participants. Interviews were conducted in a place of the participant’s choosing.

The lead (KL) and last author (KB) conducted daily debriefing sessions throughout data collection to discuss findings, amend the interview guide, refine lines of inquiry [59], and highlight where data saturation was occurring [60]. Instruments were piloted and revised during data collection. Participants were asked open-ended questions regarding their experiences living in Bhola, their health experiences, their perceptions of migration and the future and problems they identified in the area. Initial questions were designed to elicit broad personal responses and included narrative building questions. RAs probed on themes that deemed to be of relevance to the participant or were identified as important recurring themes via debriefings. Interviews were simultaneously translated and transcribed once data collection was completed.

### Data analysis

Notes from the daily debriefing sessions, reflexive and observational notes, and the interview transcripts were analyzed following (non-linearly) the six stages of Reflexive Thematic Analysis [61]. KL triangulated these three data sources and inductively developed a codebook through several stages of data familiarization, open coding and refining codes. Data were managed using NVivo Pro 12 [62].

From this codebook, we developed descriptive, content-based themes covering topics such as ‘Natural Catastrophes’, ‘Poverty’, ‘Health’, ‘Migration’, ‘Work & Education’, ‘Politics & Development’, ‘Social Life and Religion’, ‘Women and Men’. We then explored how these themes relate to mental health, as participants often described stressors and protective factors affecting their mental well-being. To deepen this analysis, we incorporated these categories into the codebook for a deductive analysis. However, we encountered challenges due to mismatches between established mental health concepts, feedback from our Bangladeshi team, and participants’ lived experiences. This led us to seek a more descriptive, culturally sensitive, and contextually grounded framework to better reflect participants’ perspectives on well-being. These considerations led us to the ‘Relational Well-being’ (RWB) framework by White [63], which emphasizes that individuals live within a interwoven, complex network of relationships and therefore experience well-being collectively. While the framework is person-centered, it acknowledges that well-being is shaped through relationships –not as resources people possess, but as connections that define who they are [33]. White highlights that people often express well-being in material or relational terms. Rather than directly discussing emotions, individuals speak about wanting enough resources to support loved ones, revealing how their emotions are expresses through actions [24,64].

White identifies three drivers of relational well-being: personal, societal and environmental. Personal drivers involve individual identity and interactions with social and material context. Societal drivers reflect socio-economic and political systems shaping well-being. Environmental drivers emphasize the connection between human well-being and planetary well-being, recognizing humans, as part of, not above, the environment [34,63]. This perspective highlights the global neglect of environmental well-being, evident in inadequate climate action [65].

To apply this framework, we coded responses as negatively influencing well-being if they reflected emotions like fear and worry (e.g., “live with fear”), hopelessness (e.g., “no hope”) or if they manifested physically through stress reactions like high blood pressure, rumination and nervousness, body pain, weakness, or tiredness (e.g., “the whole-body hurts”). Positive influences on well-being were coded when responses expressed joy or satisfaction (e.g., “feels good”) or hope, confidence and calmness (e.g., “I have hope”). Coded emotions were expressed both verbally and non-verbally. As emotions were often conveyed through actions, we used a relational lens to interpret stories – for example, the stress of earning money to support family as an expression of love (see Table S1 in S1 File: Detailed analysis description). We further contacted White to discuss our modified application of the framework and amended some of our initial labels based on her feedback (S.C. White, personal communication, October 25, 2022).

### Ethics

This study was approved by the Ethics Committee of the Medical School of Heidelberg University (S-928/2019) and the Ethics Committee of the University of Liberal Arts Bangladesh (OFR009). To participate written informed consent was necessary.

## Results

**Fig 1:**
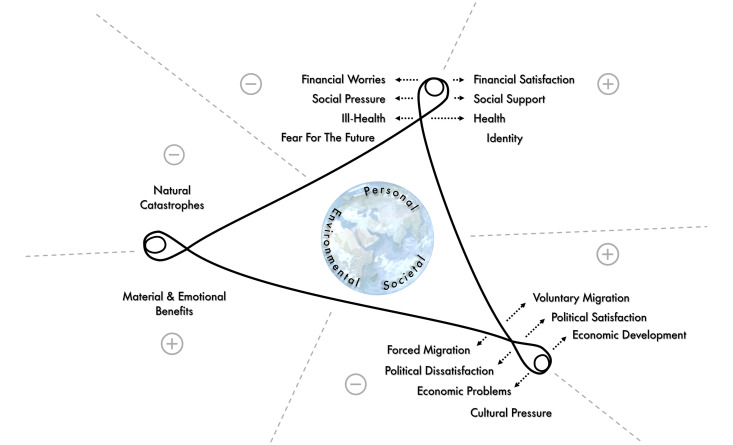
A modified Relational Well-being (RWB) framework [63]A plus sign indicates factors that positively influence well-being, while a minus sign shows those with a negative influence. Dashed lines represent factors whose roles are not definite or exclusive, highlighting that relational well-being is dynamic and constantly evolving. Arrows connect some factors to their opposing counterparts. The globe icon signifies that many factors stem from global influences, such as economic conditions, policies, and climate change. The presented factors are based on individual experiences and are not meant to be exhaustive or universally exclusive.

Participants described their well-being in relation to financial status, migration, health, social relationships, politics and economy, identity, culture and the natural environment. We present these factors as either positively or negatively influencing well-being within a modified RWB framework [24], categorized into personal, societal, and environmental drivers. We highlight that, as participants are from varying locations in Bhola, their experience of natural hazards are felt differently. For example, river erosion or flooding were major concern for those living near the river but less so for inland residents. Migration experiences also varied across participants, with migration playing a significant role in some participants’ day-to-day life, and other participants not mentioning migration at all. These differences led to diverse influences on well-being across personal, societal, and environmental drivers, as described in Fig 1.

### Personal drivers

***Negative* *factors of*** ***well-being***

#### Financial worries.

Most participants described being tense regarding their financial situation. Many reported being unable to cover living expenses and spoke of experiencing food scarcity. Insecurity of income due to unemployment or dependence from only one income source was described as increasing stress. Owing money and high education expenses were seen as even more stressful, as people felt social pressure to return debts or educate their children. Participants explained that these financial problems contributed to poor living conditions and made it “not possible to live here” (Male, 37). Some participants explained that they were unable to change their situation in any way and were therefore trapped in their current living conditions. This feeling of being trapped was mainly reported to be related to poor financial status, which itself stemmed from multiple factors such as disaster-related losses or unemployment (see Table S2 in S1 File, Q1).

*Many people have moved from here, people who do not have income or land here, they have moved. […] We do not have money [to leave], so we are still living here floating in water. (Male, 55)*

#### Social pressure and violence.

Within their social lives, participants described feeling isolated from people they would usually be close to (mostly family), saying they were lonely and lacked social support. Participants said that many families are separated because members migrated and that this led to missing family or feeling homesick. However, close relationships could also be considered stressful. Some participants felt pressured because relationships were demanding or because they were worried about or responsible for close people, mostly family members or neighbors (see Table S2 in S1 File, Q2, Q3).

*[The family is doing] not so good, they do not have any money, I am thinking how I could help them with some money. As I am elder brother, they are relying on me to help them. I have a lot of tension with them. (Male, 35)*

Some participants said that they suffered from physical insecurity and violence in the context of their social relationships. As far as personal drivers are concerned, the experience of threatened physical and mental well-being was closely interlinked with social life, and was therefore experienced at home, in public spaces and in workplaces. Participants reported physical violence like robbery, pirate attacks, sabotage, domestic violence – including fights or (sexual) abuse – and even murder. Mental violence such as verbal arguments, harassment and bullying, fraud, theft and the fear of violence or insecurity added to that burden. Such conditions fueled suspicion and reduced trust among residents (see Table S2 in S1 File, Q4, Q5).

*There is no place where husband and wife don’t hit each other. It doesn’t matter if one is rich or poor, physical abuse is there. (Female, 19)*

#### Ill-Health.

Many participants said they or their families suffered from ill-health, which was especially stressful because it endangered their families’ financial situation. The financial effects stemmed from lost income, when disabilities prevented people from working, or high treatment costs and expensive transportation to health care services. Participants would therefore only seek health care if it was affordable and locally accessible, or if the situation was critical and health care deemed absolutely necessary. However, transportation to health facilities was often difficult due to damaged infrastructure as a consequence of severe weather events.

*It [transport] is certainly a problem. Some women died during their labor pain in the middle of the way to the hospital. (Women, 25)*

Health care services could also be rejected as participants described limited trust in doctors (see Table S2 in S1 File, Q6). Participants said the causes of ill-health and disability mainly included stress and injuries or in some cases floods that, for example, caused skin diseases because of saltwater intrusion.

*I don’t feel good living here, we have lots of problems here, the tin of our house broke, due to water we have diseases. (Women, 48)*

*It’s a problem, the soil is salty. And Bhola is a floating island, it can’t be estimated when it will sink under the water. Various problems occur. My skin became darker only for salt water. There are skin problems. Allergies have started. There are allergies along with high blood pressure. This is what you see in the body. (Women, 31)*

Some participants stated that the environment in Bhola was challenging and led to fevers and colds, especially in children or workers exposed to weather events. Lack of treatment further aggravated the situation.

*We are living in such an environment where we need to stay in a trawler or boat, we have to stay in high air pressure and cold, and get ill, but no doctor can give us any treatment. (Man, 40)*

*Problems with children are that the weather here is too cold at night and too hot in the day time, so children could be suffering from cold and fever which could be a threat. (Man, 27)*

*The cold and fever increase when there is a storm. (Man,35)*

#### Fear of the future.

Participants worried about their and their family’s future. Many had to cope with the uncertainty of not knowing whether they could implement their plans because they could not rely on any kind of financial or political safeguarding. Insecurity regarding natural disasters increased that uncertainty.

*Human life is uncertain. Now I am doing well but there is no guarantee it will be same all the time. I may have worse days coming in the future! […] There is also fear in my mind because it seems that my husband’s business may be bad because the current situation [Covid-19 pandemic] is not good. […] I am afraid that this Corona Virus will finish everything all of a sudden. (Female, 31)*

Several participants mentioned plans for the government to evict them. These participants were distressed at the idea of losing their homes and wondered what would become of them (see Table S2 in S1 File, Q7).

*Don’t I have tension? What will I feed the baby, how will I raise them, how will I stay, where will I go?! We can’t stay in this area anymore, the way the river is breaking. I heard the government will kick out everyone this January. (Female, 35)*

### Positive factors of well-being

#### Financial satisfaction.

Although financial worries were a major negative factor in participants’ reported well-being, many participants also gained satisfaction from financial aspects of their lives, including those who reported suffering from financial worries. Participants appreciated financial resources such as having one or several income source(s), savings, remittances, loans and property, as this would enable them to take care of their loved ones (see Table S2 in S1 File, Q8).

*You know one thing, sister, if there is money in the family or if there is rice in the family, then there is happiness. I think men have money and women have rice in their house, so happiness and peace come later. (Female, 30)*

#### Social support.

Interpersonal relationships and close relationships, social support and joy in the company of friends and family were mentioned as positive contributors to well-being. Participants often referred to family when talking about emotional support and intimacy. Additionally, having a wider social network could also be an advantage when seeking to access services or benefits, as relationship played a big role in the distribution of goods or even government relief. Many participants felt secure and settled when they were appreciated by others and had a respected place in their community or family (see Table S2 in S1 File, Q9).

*Having family nearby is symbiotic. If they need help, we can help and vice versa. It is a huge advantage. Some relatives are living in Chittagong, by the time they can come to help I will already be dead. Long buried. But if I even have a small fever […] people from the neighboring area will come to visit. (Male, 50)*

#### Health and resilience.

We found resilience – defined by Fergus and Zimmerman as “the process of overcoming the negative effects of risk exposure, coping successfully with traumatic experiences, and avoiding the negative trajectories of risk” [66] – to allow residents to lead a life in this hazard prone region. Participants explained how they had survived under the most challenging living conditions, when having lost everything again and again due to natural catastrophes. Often, believing in God helped them to do so. They described resisting illnesses thanks to good food, hard work and a lack of diseases and acknowledged the benefits of good health (see Table S2 in S1 File, Q10, Q11).

#### Identity.

Participants described different experiences that contributed to their sense of identity and social belonging, as well as personal values and morality. Many described an underlying strong drive to live a morally righteous life, which became evident when participants emphasized the need to conduct conventional prayers, educate their children and independently take care of close (and even dead) family members, without help from others. Participants described taking responsibility as fulfilling, as they met moral expectations to contribute to their communities. Additionally, participants appreciated their education, skills, and knowledge as it helped them to master difficult situations, or they could use it to generate resources or income (see Table S2 in S1 File, Q12).

Identity was also created through a socially based sense of belonging. Participants touched on this when talking about having a special place or role within their community or family, which made them feel appreciated or proud. A strong belief in God was also a stated part of nearly all participants’ identity. Participants said faith gave them security, confidence that something will turn out positively and trust that someone was taking care of them. Faith also provided the opportunity to attribute blame if no one else could be found to assume responsibility (see Table S2 in S1 File, Q13).

*My father and mother’s grave are here... So, I said ‘no…. If the river does not destroy it, I won’t leave my parent’s grave. That’s why Allah has supported me.* […] *If there is no one to look after this grave then who will take care of them? That’s why I didn’t go. We are two brothers, and I am the eldest, that’s why I said that. (Male, 40)*

Additionally, participants expressed their future plans, hopes and dreams. They hoped for a better life for themselves, their families or their community and the development of Bhola and that they would be able to stay where they live (see, Table S2 in S1 File, Q14).

### Societal drivers

***Negative factors of well-being***

#### Forced migration.

Participants shared their perceptions on forced migration, which they had either experienced themselves or heard from other villagers. Several said they were “forced” to migrate to cities because of financial problems and hoped to find a job and earn money there. These financial problems could be related to debts they were unable to repay, unemployment or losses due to natural catastrophes such as river erosion. In cases of lost land, the unavailability of affordable land in nearby areas was also mentioned as a reason to migrate (see Table S2 in S1 File, Q15, Q16).

#### Political dissatisfaction.

Participants complained about corruption and power abuses which led to political oppression. They said they suffered from distributive injustices (such as not receiving benefits they felt they were entitled to) and deprivation that they attributed to political decisions. Participants said relief was distributed unfairly because rich people who had good political relations received more, while the others were deprived and received less or nothing at all. Moreover, participants suffered from relocation policies, where they were forced to leave their houses, and from policies associated with the Covid-19 pandemic, such as lockdowns, which resulted in job loss or income shortage.

*No small tree can grow up under the shadow of a big tree, mother. Are you understanding my words? This is a country of exploitation. One survives by killing the other one. There is nothing to do. If I want to say something I will lose my life! Can’t be said and can’t be tolerated! (Male, 78)*

#### Economic problems.

Participants criticized deficits in economic development, for example in relation to insufficient infrastructure. Roads were further degraded by weather conditions and floods, which participants said was not the case in wealthier communities (see Table S2 in S1 File, Q17). Many interviewees felt deprived regarding economic development and even taken advantage of. Participants spoke of dense settlement and overpopulation, which was stressful for many residents. Participants worried about land scarcity resulting in rising land prices, causing uncertainty as to whether they could afford to continue living in Bhola. These problems were exacerbated by river erosion and flooding, which further reduced the habitable land.

*Only the shopkeepers and owners [of fishing companies] benefit. The owners are getting rich by making us [fishermen] fools. […] Their profits are increasing day by day and we are experiencing hardship by fishing. We are fishing and we are suffering, our nets are tearing, our bodies are getting injured, our clothes are getting torn. Yet we go fishing and wish for profit as we are poor, but we have debts. (Male, 42)*

#### Cultural pressure.

Although culture was important for identity, many participants worried about how to meet cultural expectations, norms and conventions. Both men and women described feeling pressured by gender norms in different ways. Such pressures included marriage conventions and strict role allocations, with men providing for the family and women staying at home doing household work and raising children. As men had the decision-making power, women arguably suffered more from an externally determined, dependent lifestyle, which could lead to feeling treated unjust or being deprived (see Table S2 in S1 File, Q18, Q19).

*Women have to do what men say. (Female, 31)*

Other conventions touched on norms concerning morality. There was a strong expectation to live a righteous religious and autonomous life, which included performing conventional prayers, educating children and providing for one’s family independently without help from others. If participants were not able to meet these expectations, they felt social pressure, shame, and other negative emotions.

*I want them [children] to get educated and become good humans; if they get educated then we can get them married in a good place. If they are not good humans, can we do this? For this I fall sick, for this tension I fall sick. I always have this tension. Like when children don’t go to school, I feel tense. (Female, 25)*

### Positive factors of well-being

#### Voluntary migration.

The majority of participants appreciated that they had the possibility to migrate if necessary. Migration experiences included short-term, seasonal, or permanent migration for one or several family members. Many participants had experienced or heard of out-migration as a good strategy to improve financial status because migrants could diversify or increase their income, mainly through finding a job in the cities. The perception of migration as a useful strategy was widespread and many participants reported that people from their community who came back from migration were able to, for example, build a house, because their financial situation had improved. Participants therefore sometimes expressed their hope to out-migrate for a limited period, earn money and then come back to live a better life at their place of origin (see Table S2 in S1 File, Q20).

*Don’t you see these houses around, now we also have the desire if we can do the same! […] This guy has improved [pointing to neighbor’s house]. He lived abroad, made home [house] after coming back. (Male, 52)*

#### Political satisfaction & economic development.

Participants appreciated social, health and environmental security policies (e.g., the building of dams against river erosion) that benefited them. In contrast with descriptions of sources of political dissatisfaction, participants also appreciated an inclusive and supportive political system, where they felt represented, did not need to fear oppression, and benefited from equity and justice. Economically, participants appreciated progress in areas including industry, infrastructure, and employment and work opportunities, which was interlinked with economic policies. They saw these developments as safeguarding their well-being and ensuring their future in Bhola. Economic development therefore played an important role in the personal lives of participants. Their plans and dreams, their hopes to be able to stay in Bhola as well as their hopes to materially advance were closely linked to development in Bhola (see Table S2 in S1 File, Q21, Q22, Q23).

*The government has developed the roads here and that is why I am driving the ice-cream car on the roads and can have my income, and that is the most necessary income for me. There are thousands of developments that have happened here […], that is very beautiful to see, and we are happy. (Male, 45)*

### Environmental drivers

***Negative factors*** ***of* *well-being***

#### Natural catastrophes.

Environmental disasters were seen as stressful by most participants and fast-onset hazards (such as floods, storms and heavy rain) were perceived as a bigger threat than slow-onset disasters (such as river erosion or drought). Participants were anxious and worried about natural catastrophes and feared physical injury and loss, including land and livelihood loss, loss of belongings and property (houses and cattle) and loss of family members, friends, or neighbors.

These financial or emotional losses translated into critical losses with respect to people’s essential relationships. When it comes to survival, many participants rely and depend on a close social network, but also on the environment itself. Although loss was also caused by reasons such as migrated family members or business losses, most (and the most severe) experiences of loss resulted from environmental shocks. Indeed, loss caused by environmental factors was one of the most reoccurring themes within the data.

*What could be my wish? I am just waiting for my death. We do not have any other wish except death. In past days, we have dreamed of providing my children with education, but now all has got finished as the river erosion […] has taken away all our lands. Now we do not have lands, how will we survive? (Male, 26; lost his house 5–7 times in his life due to river erosion)*

However, only two participants referred to climate change here and only one of them used the actual term, while most attributed these events to “God’s will”. Other participants who recognized changes in weather patterns did not link this to climate change (see Table S2 in S1 File, Q24, Q25).

### Positive factors of well-being

#### Material and emotional benefits.

Positive factors around the environment are of a twofold nature: material and emotional. Material benefits range from natural products like fish, land to cultivate self-grown vegetables, firewood, and the more general value of a health-protecting environment, with clean water and air and open space to freely move around. Emotional benefits included place attachment with a sense of belonging or home and identity as well as a feeling of freedom. Participants expressed the hope to be able to stay in Bhola and that measures to mitigate natural disasters, such as dams, gave them a feeling of security (see Table S2 in S1 File, Q26, Q27).

*I feel good, our country, our locality, our land, we do not feel good anywhere else, beside the river is the best. (Male, 65)*

## Discussion

The rapid and far-reaching effects of climate change, particularly on climate-vulnerable populations such as in Bhola Island, Bangladesh, require innovative approaches to understanding, protecting and enhancing human mental health and well-being. Utilizing the Relational Well-being (RWB) framework [63], we explored how well-being is shaped in a population experiencing climate disaster risk. We found that well-being was negatively influenced by financial insecurities, forced migration, social pressure, political dissatisfaction, cultural expectations, and natural disasters. Meanwhile, financial satisfaction, voluntary migration, social support networks, a strong sense of identity, and attachment to place positively contribute to well-being. While many influencing factors were deeply rooted in Bhola Island’s local context, others stem from broader societal and global structures. Importantly, our findings suggest that these influencing factors are neither exhaustive nor uniformly experienced across the whole population. For example, poorer residents living along the riverbanks often perceive the natural environment as a negative influence, fearing floods and environmental hazards. In contrast, wealthier residents residing further inland or in areas protected by dams experienced their environment more positively, expressing feelings of comfort and attachment.

In Bangladesh, there are limited services available for diagnosing and treating mental ill-health [17]. Our participants did not use terms such as ‘depression’ or ‘anxiety’ and rarely referred to symptoms of clinical mental ill-health or the concept of ‘mental health’. In exchange with our Bangladeshi team members, we believe that the concept of mental health was not suitable for capturing people’s lived experiences, and that its use would not yield meaningful insights or culturally sensitive recommendations, aligning with Hayward et al. [3]. The RWB framework provided a broader perspective, enabling us to sensitively explore and incorporate what truly matters to people [27], acknowledging that individuals often describe their well-being through relationships [67]. The framework’s flexibility and its emphasis on relationships, the social and natural environment and interconnectedness allowed us to capture the complexity of various well-being influences.

Many factors influencing well-being, both positively and negatively, included a substantial material or financial component, including financial worries, migration, cultural pressure or health. Consistent with White’s findings [24,64], we observed a nuanced understanding of materiality within relationships, where material resources often serve as a means to foster and sustain social connections [16,64]. For example, the statement “If there is money in the family or if there is rice in the family, then there is happiness”, illustrates how closely financial satisfaction – enabling participants to meet basic material needs – is tied to relational well-being. This distinct understanding of materiality is also intertwined with social conventions. Studies align with our findings that social pressure and expectations to live a morally righteous life [16,22], including behaving appropriately within society and respecting gender role allocations were key ingredients for well-being. Our results also show how material needs, social relationships, and moral obligations intersect in everyday life. For example, the need to pay school fees reflect the convergence of material resources and social norms aimed at raising children properly. Moreover, morality itself emerges as a relational construct embedded in a wider social and cultural context [64]. Financial resources are essential not only to meet individual moral expectations but also uphold the family’s social standing and well-being.

Another area where the complexity of material and relational circumstances becomes evident is migration. Similar to other research, our results found migration to be perceived as either negative (forced migration) [68,69] or a positive (voluntary migration) [70], depending on individual resources and socio-demographic circumstances. Voluntary migration was described as a widespread and effective strategy for improving living conditions [71]. Other studies have framed migration as either an adaptive response to environmental changes [72–74] or a maladaptive outcome when environmental pressures exceed coping capacities [75–77]. However, portraying migration solely as adaptive is contested, as it often entails significant risks, potentially heightening vulnerability for migrants and their families [14,75–79]. Therefore, the strong positive perception of migration in our study warrants deeper examination within the broader system of well-being influences. The aspiration for elevated material status appears to drive migration in other rural communities, influences by consumerist and capitalist narratives and values, diminishing the perceived importance of social and personal costs [70]. Additionally, narratives promoting migration to meet urban labor demands [80], and success stories of migrants who gained money and social prestige [70,81–83] likely contribute to this perception. When material success is prioritized, other dimensions of well-being – such as health or identity – may become secondary, and migration is viewed as successful if it results in material gains [70]. A relational perspective on this material emphasis reveals how societal norms shape perception of materiality. Recognizing that migration involves personal, societal and environmental drivers, we advocate for a nuanced understanding that captures its multidimensional aspects [78,84]. Our findings further support existing evidence that migration decisions are often emotionally driven, shaped by local narratives, and typically made collectively by household rather than individuals [14], reinforcing the relational aspect of well-being.

Migration and climate change, both major underlying currents in the lived experience of many people in Bhola, are intricately linked to notions of ‘home’ and identity. Echoing findings from other studies, we observed that relationships with home were ambivalent [85,86]. On one hand, positive factors such as place attachment [85], social support [87,88], and identity [89,90] were closely connected to people’s understanding of Bhola as their home. On the other hand, fears of natural catastrophes [90] and desires to migrate [91] reflected negative relationships. Socio-economic and socio-ecological factors often determine which perspective prevails [92]. For rural residents, interactions and relationships with the *natural* environment are critical due to their dependence on natural resources and vulnerability to natural disasters [93]. In these communities, deep-rooted ties to land and longstanding social relationships often span generations. However, these positive influences are disrupted when individuals are forced to move to other areas [14,94].

For people in more urbanized regions, relationships with the natural and social environment may be looser, especially in populations where migration is common. For them, migration and material enhancement could outweigh positive benefits of local connections and links to the natural environment. The promises of modern capitalism might further enhance migratory desires, as shown in other semi-rural communities [70]. Still, we can see how important semi-rural or rural homes remained in the Covid-19 pandemic, when migrants returned from the cities they migrated to in order to obtain employment at their origin. In times of crises, less urban regions – such as Bhola – can serve as a backup where subsistence living and work provides safety [49]. In our study, mitigating climate change effects – by building dams to prevent river erosion – enhanced residents’ sense of security which allowed them to benefit from their relationships with people and place.

As in other studies, some participants reported changes in weather patterns, but only one participant explicitly mentioned the term climate change [95]. Aligning with evidence from Bangladesh, climate change was neither a widely understood concept [95] nor a major concern for this population. While climate change has negative effects on many of the influencing factors on well-being (e.g., ill-health, financial situation, environmental resources) [8,10,65], mitigation strategies such as dams or overall increased agricultural productivity might disguise climate change consequences [96]. The RWB framework allowed us to broadly elicit influencing factors on well-being that, in many cases, reflect common human experiences around the world, rather than being unique to our research population. Therefore, we see climate change increasing vulnerability and damaging people’s well-being without them necessarily knowing about or valuating the loss and damage it incurs [95].

Islands like Bhola are more strongly exposed to climate change consequences than inland areas and often struggle to adapt due to limited socio-economic and physical resources, with implications for human health and well-being [97]. Research from the pacific islands names heat-related illnesses, health implications of extreme weather events, water and food insecurity, migration pressure, insufficient health services, social disruption and loss of livelihoods as impacting psychological and physical well-being [98]. Coastal water pollution and coastal land inundation act as risk factors for food and water insecurity and water-, vector-, and foodborne diseases [97]. As we do, Tiatia et al. highlight culturally diverse conceptions of mental health and argue for a broad understanding of mental health and well-being, with which they find loss of identity and climate-induced migration as having negative impacts for the well-being of indigenous people in the pacific islands [99]. Other research in the pacific islands looks at how non-economic loss negatively affects mental health outcomes, via a loss of sense of place and social cohesion [100]. As in Bhola, experience of damage – especially through droughts and storms – significantly decreases well-being [101] and climate change related stress due to loss of livelihoods impairs daily lives of island inhabitants [102]. Within this context, well-being is defined collectively and holistically around indigenous knowledge [103] and multiple ecosystem services strengthen and shape relational wellbeing [104]. This also applies to islands in Norway, where natural and social capital are deeply entangled with well-being. Here, ecosystem services shape well-being through interaction and relations with the environment, even including exposure to stress or danger [105]. Moreover, research from coastal Bangladesh highlights the importance of identity, sense of place and belonging, environmental quality, agency, autonomy and strong community bonds for collective well-being [22,57]. We find that Bhola island shares many of the unique vulnerabilities of other islands and coastal areas. However, applying a relation lens when investigating what matters to people in these settings also reveals positive aspects of environmental relations, such as the fostering of identities, that can strengthen resilience and adaptation efforts.

Our study has some limitations. First, the Covid-19 safety measures prevented our research team from conducting in-person qualitative training, which may have affected the quality of the interviews. Second, conducting interviews at distance with face masks may have hindered rapport building with participants. Third, our sample was disproportionately male, as women were often restricted from participating by their partners or declined because they felt that they had nothing important to contribute. To mitigate these challenges, we held daily debriefings with the RAs, providing opportunities to discuss and resolve any issues encountered during interviews.

### Policy and research implications

A well-being approach to health could create space for climate change adaptation and risk mitigation strategies to leverage culturally appropriate, locally available resources in areas where climate hazards expose communities to continual stress. Such efforts are not a substitute for the required and urgent unmet clinical needs of individuals with mental illness. However, such an approach to designing policy may support inhabitants and build contextually realistic support mechanisms within resource poor settings where formal health services are not accessible or available.

The Gross National Happiness (GNH) Index of Bhutan is an example of approaching well-being collectively, thereby looking at social support, community vitality and relationships, environmental relations, culture, political participation and many other relational aspects that shape human well-being [29,38,106]. The GNH Index is a policy example that reflects some of the findings of this study, especially where human well-being is strongly linked to the natural and the human-made environment and protection of human health therefore has to include protection of the environment [107]. The GNH Index also stresses a core idea of well-being that is not only to focus on keeping populations alive and healthy, but also to keep them happy.

We highlight the importance of adopting open, person-centred, and holistic frameworks—such as relational well-being—to better understand lived experiences and culturally grounded definitions of well-being [3,81]. Such approaches avoid externally imposed (‘etic’) theories and instead center the voices and priorities of those most affected by climate change. Qualitative methods, in particular, offer valuable tools for exploring sensitive, context-specific meanings and for identifying what truly matters to people. Future research should build on these insights to strengthen the factors that support well-being and to mitigate those that undermine it, especially in settings facing profound environmental and social challenges.

## Supporting information

S1 File Supplemental material. (DOCX)

S2 File COREQ Guidelines. (DOCX)
