# Supplementary material for: The “most beautiful place” where “it’s not possible to live”: A qualitative study of relational well-being in an area of climate vulnerability, Bangladesh
Source: PLoS One. 2025 Sep 4;20(9):e0325972. doi: 10.1371/journal.pone.0325972 (PMC12410721; doi:10.1371/journal.pone.0325972)
Supplement: S1 File — (DOCX) [file pone.0325972.s001.docx]

# **Supplemental material**

Text S1: Interview guide

Good morning/afternoon,
Thank you for meeting with me today.

I have asked you to meet with me in the hopes of learning more about your experience and perceptions related to your health and migration. We are interested to learn any of your thoughts regarding this. Some of the questions I will ask you may not want to answer and that is fine. Remember that your participation is completely voluntary. Also please keep in mind that there are no right or wrong answers, I am interested in anything you can share with me.

As we went over in the consent, we will be taking notes and also recording our conversations so that we can accurately capture and report your views. Your names will not be recorded.

Your comments will be combined with those from other meetings with residents from this area and residents from Dhaka.

Just as a reminder our discussion will probably last around 60 minutes. Some of the questions I will ask you may not want to answer and that is fine.
Do you have any questions before we begin? May I start the recording? *[Start recording]*

| 1. Please tell me something about yourself   probe: profession/education, status in community/ family, history, health, financial status, migration experience |
| --- |
| 1. Please tell me about a normal day here in Bhola   probe: work, family, community, social activities |
| 1. Please tell me something about this area.   probe: changes in weather, population, security, personal views like/dislike, health care access, poverty, culture/religion, identity/ place attachment, community/family structure, development, jobs (new kinds of jobs, amount) |
| 1. What do you think are problems in this area? |
| 1. How do you think these issues effect men?   probe: finding work, emotional health, physical health, solutions |
| 1. How do you think these issues effect women?   probe: emotional health, physical health (maternity, pregnancy), family structure (head of household), violence (sexual, domestic), work/education, health care access, solutions |
| 1. How do you think these issues effect children?   probe: health, emotional health, education, work, safety, solutions |
| 1. What do you think will happen in this area in the future?   probe: development and its consequences (for people, environment) |
| 1. What do you think about migration from here?   probe: where people go, why, do they come back |
| 1. How do you think migration has affected this area?   probe: infrastructure, jobs, community/family structure, health care access, physical health/emotional health of residents, work distribution, remittances, neglected relatives, more work/pressure on staying people |
| 1. How do you think migration from here has affected you personally/ your family?   probe: physical and emotional health, health care access behaviour, social life change, change in work burden |
| 1. What do you think are the alternatives to migration? |
| 1. How can people from here achieve these alternatives? |
| 1. What are your plans for the future? |
| 1. Where do you think your family/community will be in the future?   probe: immediate future and long term |
| 1. What are your concerns for the future here? |
| 1. What kind of life do you think the children from this area will have?   probe: will they be here? Education / livelihoods |
| 1. What do you think of the health care in this area? |
| 1. How do you access health care? Where do you go? |
| 1. What kind of illnesses have you experienced? |
| 1. Have they changed over the past years and, if yes, why? |
| 1. If you could speak directly to a government official, what would you say to them?   probe: personal support, area support: infrastructure / facilities / government interventions |
| 1. Is there something I should have asked you that you would like to add? |

Probes refining:

Emotional/mental health: How do people feel? Depressed, anxious, angry, relieved, happy, sad, vulnerable, active, …

Community/ family structure: Which role do people play? Who decides what? What expectations do people face? Who is head of household? What is good/bad in the community?

Health care access: Ability to access health care, behaviour/opinions on health care

Table S1: Detailed analysis description

| Analysis | Description |
| --- | --- |
| Coding negative influencing factors | Evoking a wide range of ‘negative‘ emotions such as fear and worry (expressed through words like ‘very tense‘, ‘live with fear‘, ‘scared‘), helplessness and hopelessness (expressed through words like ‘feel bad/ sad‘, ‘suffer‘, be ‘in sorrow‘, be ‘in trouble‘, ‘no hope‘).  Manifesting physically through stress reactions including high blood pressure, rumination and nervousness, body pain, weakness, or tiredness (expressed though phrases like ‘ill and tired ‘, ‘the whole-body hurts ‘, ruminating ‘many nights while sleeping ‘, having ‘heart problems for thinking about business ‘) |
| Coding positive influencing factors | Evoking a wide range of ‘positive‘ feelings, such as joy, happiness or satisfaction (expressed through words such as ‘happiness‘ , ‘peace‘, something ‘feels good‘, something ‘was good on all sides‘), hope, confidence and a calm, feel-good state of being (expressed though phrases like ‘I have hope‘ or through talking about dreams and wishes) |
| Expression of emotions | Verbally (words, tone, volume, expression of emotions like crying and laughing) and non-verbally (gestures, facial expressions) |
| Cultural lens | Participants often expressed emotions through talking about actions, so we interpreted participants’ stories through a relational lens to capture their ways of expressing feelings. An example would be participants talking about the stress of earning enough money to provide for their families as an expression for their affection and love. |

Table S2: Additional quotes

| Number of Quote | Quote |
| --- | --- |
| Q1 | *We wouldn’t have stayed here. We would have tried to build a house; we wouldn’t stay in this house and drink this water. We would have moved out and another helpless family would have come here. Now, we can’t live in this place, this is a tension in my mind. (Female, 25)* |
| Q2 | *Grandmother didn’t love us much, and for not getting love I started feeling bad. Then I asked my mother: “Are we born with little luck, why did you give us birth? We see people, they get so much love from their mother and father, they get love from all their aunts and uncles. We didn't get that, what are we born with on this earth?” Then my parents made me understand that we are born in a poor family. (Female, 18)* |
| Q3 | *The day before yesterday I talked to my mother. Mom asked, "O boy, how long have you been here?" It's been a month and a half since I visited my village. But, listening to my mother, it seems that it has been an era. My own tears are coming when I hear my mother's words. My mother wants to see me, but I can't see her. It feels very bad then. (Male, 20)* |
| Q4 | *They [dacoits] shouted like anything… they… they...they brought their boggy [sharp weapon], they hit them with their boggy...they told them not to shout, whoever it was didn’t scream; they tied both of us and whatever things we had they took them. […] In our house at least ten men entered. With them I will fight and catch them, later I thought they will catch my family that’s why I didn’t take action. as I didn’t take any action, they took whatever they got...as their wish. (Male, 40)* |
| Q5 | *We are all alone, they are after me for we are left behind, we don’t have anyone. There are many people who team up; gossip how to ruin someone. About a year ago there was a woman who tortured us; they used to take away our cloths at night, she used to break our stove. They used to urinate inside our stove, that’s how that lady used to torture us. (Female, 18)* |
| Q6 | *The doctors are like the butcher. Like I am having a tonsil that has been given by the Almighty, but for having treated for that the doctors make a deal of like 25 to 30 thousands to get operated from Dhaka and along with other expenditures it will cost like 60 to 70 thousand taka. They will not care about us, how we will manage the money. They just directly say the amount of money is like 25/ 30,000 and if we cannot manage that then we will not get the treatment. (Male, 42)* |
| Q7 | *The government will evict us in January. We need a place. If we are evicted, where we will go, where will we stay, where we will go? It’s already very difficult to eat and drink this way. (Female, 20)* |
| Q8 | *If you give loan to the women, they can raise domestic animals; they can also look after family and finances. (Male, 55)* |
| Q9 | *This area is good, […] we all live together. It’s like, if I need anything, beside our house there is “mami” [calls her neighbour “aunty”], people living in this area are like our relatives. We all stay together, in one place and in one house. For any problem I come here; that day I came here to “mami”, told her that we don’t have onions, took few onions or if I need salt, oil I take it from her. And when we bring those for our house, we give them back. This is how it works. (Female, 25)* |
| Q10 | *My father and mother died in the floodwaters of the 1970s. At that time, 12 members of our family died together. I lived alone here. I was studying in class 7 during that time. I have been farming since then. I got married in 1984. I have been here ever since. Now I have 4 daughters and 4 sons. (Male, 65)* |
| Q11 | *If human health is good, then everything feels good. Nothing in the world feels good without good health. (Male, 20)* |
| Q12 | *I learn to work, now I am learning to stitch. By learning this in the future I could earn a job with it. […] I brought a scissor [she smiled], I asked mother to bring me yarn, people do it; just because we are poor… so happy about it. People also feel happy about it. (Female, 18)* |
| Q13 | *I think that Allah didn’t feed me today he will tomorrow. If not tomorrow, then the day after tomorrow. I don't feel bad for that, If Allah gave tofique [blessings] then I will get and if not then I will not, this is what I believe. (Female, 19)* |
| Q14 | *It is better if a mill factory is built. Because the people who go to Dhaka would not have to go so far for their work. By working here, they could stay with the people closest to them. (Female, 31)* |
| Q15 | *Many ran away after they racked up a lot of loans. They try to come back here but they can’t pay back the loans. If they come back the loan sharks will hurt them. (Male, 22)* |
| Q16 | *[They were] forced to leave. No one leaves their homeland willingly. (Male, 18)* |
| Q17 | *Weather is not good. […] The water rises, the roads collapse, even when the weather is hot. Men cannot go out to work in time because of the bad roads. We cannot go to the pond because the road is muddy. The road becomes muddier because of herding cattle. Women have a lot of work to do in the pond but cannot go for the mud. (Female, 25)* |
| Q18 | *We are the poor people. In order to protect self-respect, I married away the girl early. [...] Many people talk a lot when they [daughters] are a little older. (Male, 52)* |
| Q19 | *I don’t like women coming out of the house and walking around. It is against the rule of Islam. (Male, 50)* |
| Q20 | *People move to Dhaka for doing jobs as here is scarcity of jobs. They go there as there are more opportunities in Dhaka. As there is a saying, “Money flies in Dhaka”. (Male, 45)* |
| Q21 | *Before they could not go out, because the roads used to be under water from the tide, they used to go by boats, so there was fear and lack of security. Now things are not the same due to the dam. (Male, 63)* |
| Q22 | *Work in the area has increased. Now the embankment is developing so people have the opportunity to work there. (Female, 48)* |
| Q23 | *There are so many arrangements here […] which were not available previously. If I can earn here, then why should I move to Dhaka with my family? (Male, 50)* |
| Q24 | *During that time people became fierce. Their house is beside the river, they get scared as they live by the river, where will we go? Who will give us shelter […] when the water... O gosh! can’t talk about the storm and wind, can we survive? - There is no guarantee. (Female, 18)* |
| Q25 | *If Allah had given ability, I would not have stayed here. I would not have seen the wind and storm. Those who have trees and houses in the highlands have no fear. We have nothing here, all of us like to run away. (Female, 25)* |
| Q26 | *We live in a village, and for us this place is more beautiful than other places. No other place is more beautiful than our village to us. (Male, 51)* |
| Q27 | *We have fresh food, we grow our own, and we eat it. […] That is good for us. But city people are not like that. Allah has given them money but they eat frozen food, food that is grown in the laboratory. […] We have an open place, children swim here, there are fresh fish that they eat. But city people eat fish that are sold by the fishermen in the market and keep them into the fridge which are not as fresh as ours. (Male, 45)* |
