# Supplementary material for: The “most beautiful place” where “it’s not possible to live”: A qualitative study of relational well-being in an area of climate vulnerability, Bangladesh
Source: PLoS One. 2025 Sep 4;20(9):e0325972. doi: 10.1371/journal.pone.0325972 (PMC12410721; doi:10.1371/journal.pone.0325972)
Supplement: S2 File — (DOCX) [file pone.0325972.s002.docx]

## Table 1: COREQ Guidelines: 32-­‐ITEM CHECKLIST ^1^

| No. Item | Guide questions/description | Reported on Page # |
| --- | --- | --- |
| Domain 1: Research team  and reﬂexivity |  |  |
| 1. Interviewer/facilitator | Which author/s conducted the  interview? | STR, RI, JD |
| 2. Credentials | What were the researcher’s credentials? | 8 |
| 3. Occupation | What was their occupation at the time of  the study? | 8 |
| 4. Gender | Was the researcher male or female? | 8 |
| 5. Experience and training | What experience or training did the  researchers have? | 8 |
| 6. Relationship with participants established | Was a relationship established prior to study commencement? | 9 |
| 7. Participant knowledge  of the interviewer | What did the participants know about  the researcher? | 9 |
| 8. Interviewer  characteristics | What characteristics were reported  about the interviewer/facilitator? | 8 |
| Domain 2: study design |  |  |
| 9. Methodological  orientation and Theory | What methodological orientation was  stated to underpin the study? | 6 |
| 10. Sampling | How were participants selected? | 7 |
| 11. Method of approach | How were participants approached? | 7 |
| 12. Sample size | How many participants were in the  study? | 7, 8 |
| 13. Non-participation | How many people refused to participate  or dropped out? Reasons? | 29 |
| 14. Setting of data collection | Where was the data collected? | 6, 7 |
| 15. Presence of non-­‐  participants | Was anyone else present besides the  participants and researchers? | - |
| 16. Description of sample | What are the important characteristics  of the sample? | Table 1 |
| 17. Interview guide | Were questions, prompts, guides provided by the authors? | 8 and Supplement Material |
| 18. Repeat interviews | Were repeat interviews carried out? | no |
| 19. Audio/visual recording | Did the research use audio or visual recording to collect the data? | 8 |
| 20. Field notes | Were ﬁeld notes made during and/or after the interview? | 8 |
| 21. Duration | What was the duration of the interviews? | 8 |
| 22. Data saturation | Was data saturation discussed? | 7 |
| 23. Transcripts returned | Were transcripts returned to  participants for comment and/or correction? | No |
| Domain 3: analysis and  ﬁndings |  |  |
| 24. Number of data coders | How many data coders coded the data? | 9 |
| 25. Description of the  coding tree | Did authors provide a description of the  coding tree? | 9 |
| 26. Derivation of themes | Were themes identiﬁed in advance or  derived from the data? | 9-11 |
| 27. Software | What software, if applicable, was used to manage the data? | 9 |
| 28. Participant checking | Did participants provide feedback on  the ﬁndings? | No |
| 29. Quotations presented | Were participant quotations presented to illustrate the themes/ﬁndings? Was  each quotation identiﬁed? | 10, 11, Supplemental material Table 1 |
| 30. Data and ﬁndings  consistent | Was there consistency between the data  presented and the ﬁndings? | 11-24 |
| 31. Clarity of major themes | Were major themes clearly presented in the ﬁndings? | 11-24 |
| 32. Clarity of minor  themes | Is there a description of diverse cases or  discussion of minor themes? | 11-24 |

References

1. Tong A, Sainsbury P, Craig J. Consolidated criteria for reporting qualitative research (COREQ): a 32-item checklist for interviews and focus groups. *Int J Qual Health Care*. 2007;19(6):349-357. doi:10.1093/intqhc/mzm042.
